# Recent advances in battery characterization using in situ XAFS, SAXS, XRD, and their combining techniques: From single scale to multiscale structure detection

**DOI:** 10.1002/EXP.20230056

**Published:** 2023-11-20

**Authors:** Weidong Cheng, Mengyuan Zhao, Yuecheng Lai, Xin Wang, Huanyan Liu, Peng Xiao, Guang Mo, Bin Liu, Yunpeng Liu

**Affiliations:** ^1^ College of Materials Science and Engineering Qiqihar University Qiqihar China; ^2^ Beijing Synchrotron Radiation Facility, Institute of High Energy Physics Chinese Academy of Sciences Beijing China; ^3^ Chinese Academy of Sciences University of Chinese Academy of Sciences Beijing China; ^4^ State Key Laboratory of Heavy Oil Processing, The Key Laboratory of Catalysis of CNPC, College of Chemical Engineering China University of Petroleum Beijing China; ^5^ State Key Laboratory of Chemical Resource Engineering, College of Chemistry Beijing University of Chemical Technology Beijing China

**Keywords:** batteries, combining technique, small‐angle X‐ray scattering, structure detection, X‐ray absorption fine structure, X‐ray diffraction

## Abstract

Revealing and clarifying the chemical reaction processes and mechanisms inside the batteries will bring a great help to the controllable preparation and performance modulation of batteries. Advanced characterization techniques based on synchrotron radiation (SR) have accelerated the development of various batteries over the past decade. In situ SR techniques have been widely used in the study of electrochemical reactions and mechanisms due to their excellent characteristics. Herein, the three most wide and important synchrotron radiation techniques used in battery research were systematically reviewed, namely X‐ray absorption fine structure (XAFS) spectroscopy, small‐angle X‐ray scattering (SAXS), and X‐ray diffraction (XRD). Special attention is paid to how these characterization techniques are used to understand the reaction mechanism of batteries and improve the practical characteristics of batteries. Moreover, the in situ combining techniques advance the acquisition of single scale structure information to the simultaneous characterization of multiscale structures, which will bring a new perspective to the research of batteries. Finally, the challenges and future opportunities of SR techniques for battery research are featured based on their current development.

## Graphical abstract

Advanced characterization techniques contribute to revealing and clarifying the chemical reaction processes and mechanisms inside the batteries. This review comprehensively overviews the recent advances in battery characterization using in situ synchrotron X‐ray absorption fine structure (XAFS), small‐angle X‐ray scattering (SAXS), X‐ray diffraction (XRD), and their combining techniques.

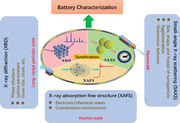

## 1 INTRODUCTION

With the development of human economy and society, environmental pollution and energy crisis are increasing.^[^ ^1^, ^2^, ^3^ ^]^ Clean and renewable energy storage and conversion systems have to rapidly develop.^[^ ^4^, ^5^, ^6^ ^]^ Typically, the electrochemical energy storage technology,^[^ ^7^, ^8^ ^]^ including batteries and supercapacitors, receives much attention due to its high energy density, long service life, and environmental friendliness.^[^ ^9^, ^10^, ^11^, ^12^, ^13^, ^14^ ^]^ Between the two components, it is well known that batteries,^[^ ^15^ ^]^ such as lithium‐ion batteries (LIBs),^[^ ^16^, ^17^ ^]^ sodium‐ion batteries (SIBs),^[^ ^18^, ^19^ ^]^ potassium‐ion batteries (PIBs),^[^ ^20^ ^]^ zinc‐ion batteries (ZIBs),^[^ ^21^, ^22^ ^]^ aluminum‐based batteries,^[^ ^23^ ^]^ etc.^[^ ^24^, ^25^ ^]^ are the major components of energy storage and conversion systems and have attracted much interest from global researchers.

Although the electrochemical energy storage technologies or batteries are a focal point of continuously growing research and development work worldwide, many competitive factors, such as performance and safety, stability and activity, capacity and cyclability, etc. in energy materials limit their advances and cannot be undervalued. The typical challenges lithium‐based batteries are that the inevitable capacity degradation and increased internal resistance of LIBs during operating conditions (cyclic charging and discharging,^[^ ^26^ ^]^ extreme hot and cold^[^ ^27^, ^28^ ^]^). New battery systems,^[^ ^29^, ^30^, ^31^ ^]^ which use high‐capacity metal anodes (such as Li, Zn, and Al) and chalcogen cathodes (such as O_2_, S, and Se), are developing to meet growing energy demand.^[^ ^32^, ^33^ ^]^ For example, the Li–S battery can provide about 2.15 V working voltage and an average 2600 Wh kg^−1^ theoretical specific energy.^[^ ^34^ ^]^ However, on the Li metal anode, the dendrites and solid electrolyte interphase (SEI) formation remain its main challenge in the reversibility of Li plating and stripping.^[^ ^13^ ^]^ The next‐generation secondary nonlithium battery system with high specific energy is also explored for future applications. SIBs^[^ ^19^, ^35^, ^36^ ^]^ and PIBs^[^ ^37^ ^]^ have a similar electrochemical performance to LIBs due to the same main group as lithium, abundant sodium and potassium resources, and low cost. But the anode materials of SIBs generally suffer from serious volume expansion and capacity loss, which affect the cycle life and energy density. Controlling the thickness and chemical composition of the SEI film in SIBs is an important issue.^[^ ^35^ ^]^ For the PIBs, its graphite anode usually exhibits a large volume change of ≈60% during the K^+^ insertion/reinsertion process.^[^ ^38^ ^]^ The nanocrystal‐growth habit generally causes low tap density and volumetric energy density for Prussian blue materials.^[^ ^37^ ^]^ For the ZIBs^[^ ^39^, ^40^ ^]^ with high theoretical capacity (about 820 mAh g^−1^) and low cost,^[^ ^41^ ^]^ the Zn metal anode faces a serious problem of Zn irreversibility due to the nucleation and growth of Zn dendrite determined by both thermodynamics and kinetics of ZnO passivation.^[^ ^42^, ^43^, ^44^ ^]^ Besides, the aqueous ZIBs have been tackling the dendrite formation and Zn corrosion issues of the Zn metal anode. Moreover, the insertion and removal of ions such as H^+^ and Zn^2+^ can cause lattice expansion/contraction and irreversible phase transition, resulting in the formation of by‐products such as Zn*_x_* MnO_4_, MnOOH, ZnMn_3_O_7_·3H_2_O, and Zn_2_Mn_3_O_8_^[^ ^45^ ^]^ as well as cracking and dissolution of electrode materials. The anode/cathode electrolyte interphase can also react with aqueous electrolyte and H^+^ during cycling, resulting in the formation of loose and porous layers.^[^ ^40^, ^46^ ^]^

Revealing and clarifying the chemical reaction process and formation mechanism inside the batteries will bring a great help to solve the problems mentioned above and improve the performances of batteries. For example, by revealing the SEI^[^ ^47^, ^48^ ^]^ formation mechanism, one can regulate the morphologies, predict the degradation and design novel‐nanostructured electrode materials with higher specific capacities; disclosing the dendrites^[^ ^49^ ^]^ growth process, one can avoid to puncture the diaphragm, trigger internal short circuits. Anyway, in‐situ characterization of the structures with different levels for battery materials is always the key step, among which the X‐ray techniques based on synchrotron radiation^[^ ^50^ ^]^ (SR) play key roles and have extensive applications.^[^ ^51^, ^52^, ^53^, ^54^ ^]^ X‐ray absorption and scattering methods in SR techniques, mainly including X‐ray absorption fine structure (XAFS) spectroscopy, small‐angle X‐ray scattering (SAXS), wide‐angle X‐ray scattering (WAXS), and X‐ray diffraction (XRD), are essential to characterize material structures. XAFS is sensitive to the short‐range structure and can be used to obtain the atomic neighbor structure inside the materials, such as oxidation state, coordination number, and distance of the specific element.^[^ ^52^, ^55^, ^56^ ^]^ Through XAFS technique, the atomic scale structural changes of battery materials during charging and discharging can be detected and then the performance and lifespan of the battery can be optimized. SAXS is sensitive to the fluctuation of electron density inside the materials, and is a powerful tool to study the nanoscale structure^[^ ^57^ ^]^ of materials, such as size, shape, fractal, agglomeration, and orientation behavior, etc., of nanoparticles. Through SAXS technique, it is possible to understand the nanoparticle aggregation in battery materials, the pore structure of electrode materials, and the diffusion behavior of electrolyte. This information is crucial for designing and optimizing the structure and performance of electrode materials. WAXS or XRD is sensitive to the long‐period ordered structure of materials,^[^ ^58^, ^59^ ^]^ which can quantitatively describe the crystal structure information, such as lattice parameters, grain size, strain, etc., of crystalline phases. Through XRD technique, it is possible to understand the impact of these factors on battery performance. SR‐based XAFS, SAXS, and XRD techniques are truly capable of understanding the basic structure knowledge of battery materials at atomic/molecular, nano, micro levels, and contribute to the future development of novel or high‐performance energy materials.

In the current existing reviews about in situ/operando SR techniques for battery research, researchers often focus on one experimental technique and structural study with a single scale. Fehse et al.^[^ ^60^ ^]^ reviewed the in situ X‐ray spectroscopy for revealing the functional mechanisms in batteries; Ren et al.^[^ ^61^ ^]^ summarized the SR scattering technique for rechargeable battery research. Though Song et al.^[^ ^62^ ^]^ reviewed many SR‐based characterization techniques, such as XRD, X‐ray photoelectron spectroscopy (XPS), XAFS, XCT, etc., they just focus on the uncovering of the local structure and chemistry of metal–air batteries. Herein, we focus on the recent applications of the three in situ XAFS, SAXS, and XRD as well as their combining techniques in detecting hierarchical structural information of electrode materials from single scale to multiscale, from atomic/molecular level to nanoscale to micro level and to the three combining. From the perspective of the structural scale of battery materials, this review contributes to revealing and clarifying the chemical reaction processes and mechanisms inside the batteries, aiming to provide new insights on how to improve the energy storage performance of batteries and promote the realization of renewable energy and sustainable development. Also, the challenges and future opportunities of SR techniques for battery research will be also featured based on their current development.

## 2 SYCHROTRON RADIATION X‐RAY ABSORPTION AND SCATTERING TECHNIQUES

Synchrotron radiation (SR)^[^ ^63^ ^]^ X‐ray is the electromagnetic waves emitted along the tangent direction of the electron orbit when high‐speed electrons pass through the bending magnet in circular accelerator. Compared with traditional laboratory X‐ray source, SR X‐rays have multitudinous excellent properties, such as high brightness, high collimation, high stability, wide band coverage, high polarization, and pulse time structure.^[^ ^64^, ^65^, ^66^, ^67^ ^]^ SR X‐ray characterization techniques have also many advantages, such as high time/space/energy resolution, in‐situ/real‐time detecting, dynamic evolution investigation, etc., which are indispensable in contemporary scientific research.^[^ ^68^, ^69^, ^70^ ^]^

**FIGURE 1:**
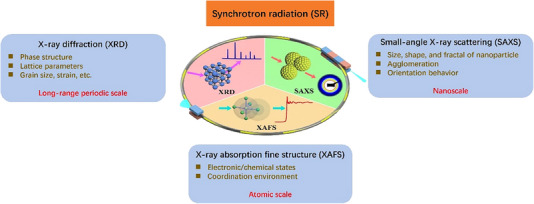
Schematic illustration of the representative synchrotron radiation X‐ray absorption fine structure, small‐angle X‐ray scattering, and X‐ray diffraction techniques.

The interaction of X‐rays with matter is mainly divided into scattering and absorption. Here, we will be focusing on the most widely used absorption and elastic scattering methods,^[^ ^71^ ^]^ that is, X‐ray absorption fine structure (XAFS) spectroscopy, small‐angle X‐ray scattering (SAXS), and X‐ray diffraction (XRD), aiming to characterize structures from atom/molecule scale to nanoscale to micron‐scale for in‐situ/operando rechargeable battery materials, as shown in Figure 1. XAFS measures the variation curve of the X‐ray absorption coefficient with X‐ray energy.^[^ ^50^ ^]^ Typically, XAFS can be divided into X‐ray absorption near‐edge structure (XANES)^[^ ^72^ ^]^ and extended X‐ray absorption fine structure (EXAFS).^[^ ^73^ ^]^ XANES covers the range from about 20 eV before the absorption edge (AE) to about 50 eV behind the AE, which is mainly originated from the single‐electron multiple scattering effects of the inner shell photoelectrons. XANES was used to determine the oxidation states, components, and coordination geometry configurations of the absorption elements in the material by comparing with reference materials.^[^ ^74^ ^]^ EXAFS has a broad energy range and is about 50–1000 eV behind the AE, which derives from the single electron single scattering effect of the inner photoelectron. EXAFS can reveal the coordination environment of the absorption element, such as coordination‐atom types, numbers, and distances by fitting.^[^ ^75^ ^]^ SAXS and XRD discussed in this review belong to the category of elastic scattering techniques, in which X‐ray photons do not experience energy loss after scattering.^[^ ^76^ ^]^ Compared to XAFS, SAXS detects structures over a larger length scale, ranging from 1 nm to several hundred nanometers. SAXS is particularly powerful in characterizing structural morphology (size and shape), especially at the nanoscale. In addition to traditional SAXS techniques, SAXS experiment methods also include ultra‐small angle X‐ray scattering (USAXS),^[^ ^77^ ^]^ anomalous SAXS (ASAXS),^[^ ^78^ ^]^ and grazing‐incidence SAXS (GiSAXS).^[^ ^79^ ^]^ USAXS can detect particle structure up to a few micrometers. ASAXS utilizes variations in specific elements to obtain information about the distribution of resonant atoms in a sample. GiSAXS is a powerful tool for analyzing the morphology and distribution of islands on substrates or buried particles. XRD is the primary method for studying the periodic structure of materials, such as lattice parameters, grain size, strain, etc. For SR‐XRD, the energy or wavelength of incident X‐ray can be selected in a wide range, and the data with a high signal‐to‐noise (SNR) ratio, high angular resolution, and high energy resolution^[^ ^80^ ^]^ can be obtained quickly and accurately. It should be mentioned here, in fact, there is no fundamental difference between WAXS and XRD, the basic principles of the two are the same. The core differences between WAXS and XRD are the detector used and the range of recorded data. Generally speaking, WAXS patterns are recorded by 2D area detector using transmission method, and XRD data is collected by 1D or point detector using reflection method; and the range of recorded data for XRD (5–130°) is larger than WAXS (5–80°). XRD is a special type of scattering, relatively more representative and universal in SR techniques used to study batteries. Furthermore, the advanced time‐resolved in situ SR XAFS, SAXS, and XRD techniques can real‐time monitor the structural evolution of electrode materials during electrochemical reactions, which is crucial for uncovering the reaction processes and mechanisms.^[^ ^81^, ^82^, ^83^ ^]^ From single experiment to combined experiment, from single scale to multiscale structure detection, the applications of in situ XAFS, SAXS, XRD and their combining techniques in battery characterization will inevitably provide important guidance for the controllable preparation and performance improvement of battery materials.^[^ ^61^, ^84^, ^85^ ^]^

In the following sections, we will provide a few recent examples to demonstrate the applications of these techniques in structural characterization of battery materials when operating in‐situ or operando measurements which elucidate the battery working mechanisms and performances.

## 3 APPLICATION EXAMPLES OF BATTERY STRUCTURE DETECTION

### 3.1 XAFS for revealing atomic local structure

XAFS is essential to reveal the atomic local structures in materials. It can be used to study the valence states and the coordination environments (the atom type, atom number, and coordinated bond‐length) of metal elements in batteries. The samples used for XAFS experiments can be solid in the form of powder or film and liquid. Different samples require different preparation method, for instance, the powder samples often were processed by smearing tape, pressed tablets. Generally, XAFS can be divided into two modes, namely, transmission mode and fluorescence mode, based the content of the element to be measured. The former is for the mass ratio of the element to be measured above 10%; the latter is for the mass ratio of the element to be measured below 10% and above 0.01%. The high‐performing front‐rear gas ionization chamber detectors were used to collect the data. Placing the in‐situ battery devices between two detectors and changing the X‐ray energy by rotating the monochromator and conducting energy scanning by software, the X‐ray absorption spectrum is obtained. XAFS also has some drawbacks, it can only be used to analyze neighbor structures of the absorbed atoms, and even some coordinated atoms cannot be distinguished (such as carbon, nitrogen, and oxygen) and needs to rely on other characterization results to analyze the data. In this section, a few typical recent examples of in situ XAFS technique in battery electrode materials will be discussed, aiming to illustrate how the XAFS elucidate the electrochemical reaction process and mechanism in atomic scale.

Since it was first used in the field of batteries by McBreen et al.^[^ ^86^ ^]^ in 1988, the number of XAFS applications have rapidly grown with the increasing of in situ and operando measurements. In general, most researchers use transition metal (e.g., Mn, Fe, Co, and Ni) oxide materials as electrode materials, which undergoing the insertion reactions for cathode materials and the conversion reactions for negative electrode materials, respectively.^[^ ^60^ ^]^ Su et al.^[^ ^87^ ^]^ explored the conversion reaction mechanism of the CoMn_2_O_4_ electrode using in situ XAFS technique. It was found that both Co^2+^ and Mn^3+^ in CoMn_2_O_4_ were reduced to metallic Mn and Co during the first cycle process, while metallic Mn and Co were oxidized to CoO and MnO during the charging process; from the second cycle of charge and discharge, the conversion reaction was described as the transformation of CoO and MnO into metallic Co and Mn, and vice versa; furthermore, no changes were observed in the XANES and EXAFS spectra of Mn and Co K‐edges during the discharge process, indicating that there was no valence state and phase transition evolutions in CoMn_2_O_4_ electrode material, which implicitly explained the formation of SEI on the surface of CoMn_2_O_4_ electrode.^[^ ^87^ ^]^ Ding et al.^[^ ^88^ ^]^ investigated the particular local environment changes of a novel Sn_4_P_3_/graphene composite anode material in LIBs with superior capacity and cycling performance (651 mAh g^−1^ after 100 cycles) during lithiation and delithiation processes by in situ EXAFS. The results show that in the first two cycles, the crystalline Sn_4_P_3_ was completely transformed into an amorphous phase, which participated in reversible conversion and alloy reactions; XAFS characterization helps to uncover the mechanism of highly reversible tin phosphides and offers insights for enhancing the capacity and cycle life of conversion and alloying materials. Also, Gunaydin et al.^[^ ^89^ ^]^ investigated the effects of boron substitution on the crystal and electronic structure properties of LiFeO_2_ materials. XAFS results indicate that the high electronegativity of boron atoms causes neighboring Fe─O bonds to relax and leads to the decay of iron oxidation states, giving a good guidance of achieving better performance values by reducing excess oxygen in the material.

**FIGURE 2:**
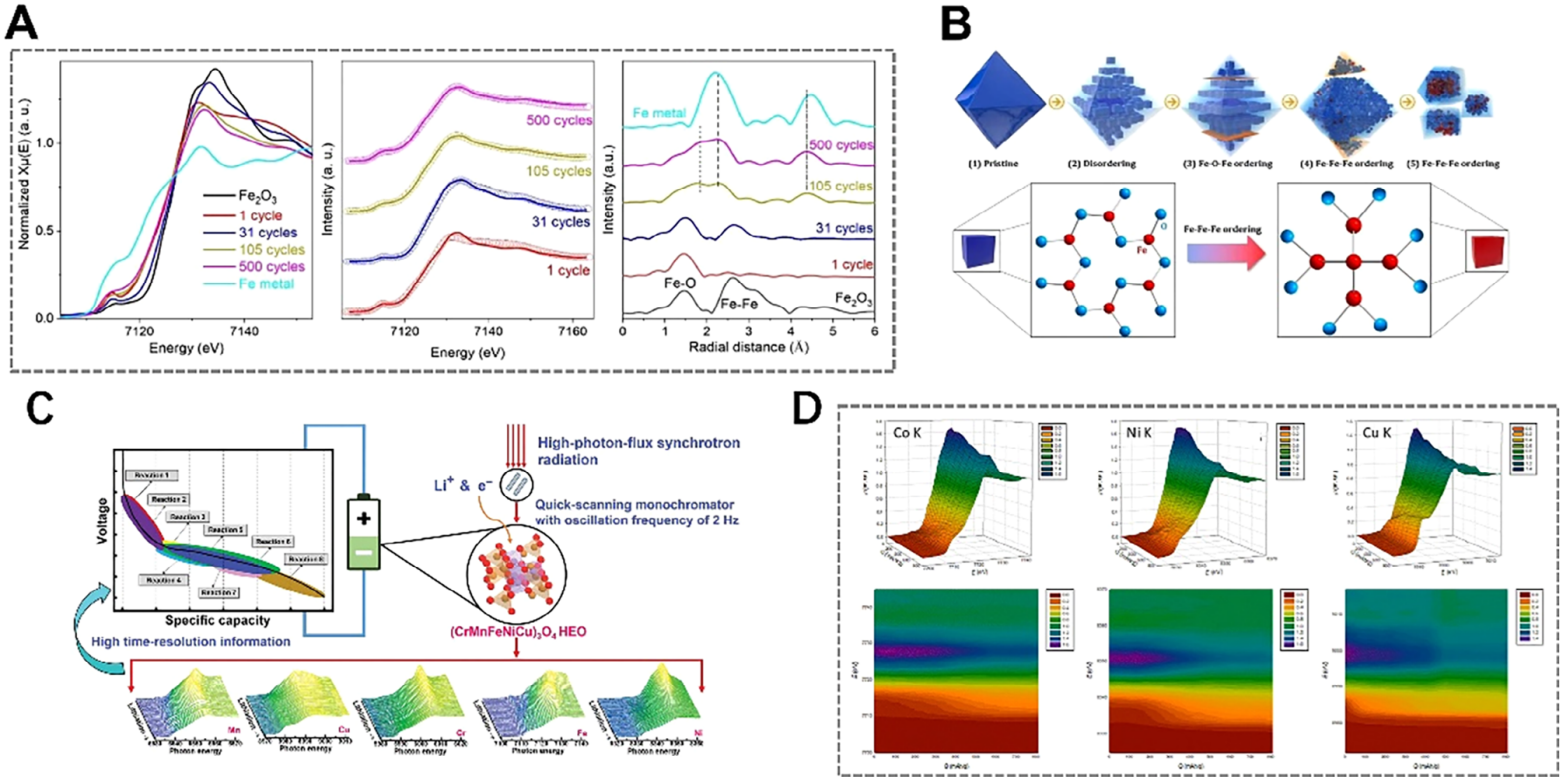
In situ X‐ray absorption fine structure technique reveals the atomic neighbor structures of different metal‐based electrodes after different cycles. (A) XANES data (left), the corresponding linear combination fit (middle) and the Fourier transform of EXAFS data (right) of Fe_2_O_3_ electrode and (B) the related schematic diagram explaining morphological and structural evolution during the cycling process. Reproduced with permission.^[^ ^90^ ^]^ Copyright 2021, Elsevier. (C) in‐situ QXAFS study of (CrMnFeNiCu)_3_O_4_ HEO electrode. Reproduced with permission.^[^ ^91^ ^]^ Copyright 2022, Wiley. (D) In situ Co, Ni, and Cu K‐edge XANES spectra in a working HEO battery. Reproduced with permission.^[^ ^92^ ^]^ Copyright 2020, American Chemical Society.

To further investigate the neighbor structure information in batteries, herein, the ex/in situ Fe K‐edge XAFS results of Zhang,^[^ ^90^ ^]^ Luo,^[^ ^91^ ^]^ and Ghigna^[^ ^92^ ^]^ et al. were clearly illustrated. According to the XANES spectra (Figure 2A, left), the absorption‐edge moves towards the lower energy direction with cycling, which corresponds to the lower oxidation state of Fe ions. The XANES data of the cycled electrode can be well fitted by a linear combination of Fe foil, FeO, Fe_3_O_4_, and Fe_2_O_3_, except for the first cycle (Figure 2A, middle). The poor fitting adaptability of the electrode after the first cycle indicates that the local structure of Fe at this site is different from the local structures of these known Fe species. After 31 cycles, a large amount of Fe_3_O_4_‐like Fe species appeared and then gradually transformed into Fe^0^‐like species in the subsequent cycling process. Surprisingly, further cycles (i.e., 105 cycles and above) introduce several additional peaks at larger radial distances, as well as a continuous shift of the two main signals corresponding to Fe─O and Fe─Fe bonds^[^ ^90^ ^]^ (Figure 2A, right). Based on the XAFS results, the morphological and structural evolutions during cycles are depicted (Figure 2B). These results confirm that XAFS technique contributes to better understanding the observed capacity increase during the cycling of the conversion electrode. The lithiation/decay mechanism can be well revealed by in situ quick‐scanning XAFS (QXAFS). The valence/coordination state changes, multiple transition steps, reversibility, redox sequence, and redox overpotential of non‐cobalt spinel (CrMnFeNiCu)_3_O_4_ high‐entropy oxide (HEO) electrode material were investigated^[^ ^91^ ^]^ (Figure 2C). The results show that during lithiation, Cu^2+^ and Ni^2+^ cations were reduced to Cu and Ni, and Mn^2+/3+^ and Fe^2+/3+^ cations were transformed into metal phases through two‐step conversion reactions with MnO and FeO as intermediate substances, respectively, and Cr^3+^ ions were first reduced to CrO and then to Cr; during decay, the oxidation of Mn occurred before that of Fe. Besides, in‐situ XAFS also was used to explore the lithium/alloying mechanism of the transition metal‐based high‐entropy oxides (TM‐HEO). Ghigna et al.^[^ ^92^ ^]^ conducted an operando XAFS study on TM‐HEO‐based anodes for LIBs during the first lithiation/delithiation process (Figure 2D). This kind of material exhibited a high specific capacity of over 600 mAh g^−1^ at 0.1C and Coulombic efficiency approach to 1. The advanced XAFS disclosed a complicated charging mechanism, triggering the conversion reaction below 1.0 V.

**FIGURE 3:**
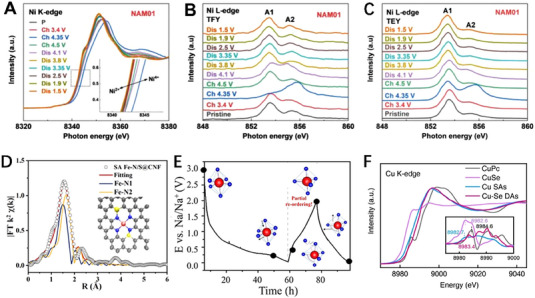
In situ X‐ray absorption fine structure technique helps clarify the chemical mechanisms of electrode materials in the process of electrochemical reactions. (A) Ni K‐edge, (B) TFY Ni L_3_‐edge, and (C) TEY Ni L_3_‐edge XANES spectra of Na_0.6_Ni_0.25_Al_0.1_Mn_0.65_O_2_ under the reaction. Reproduced with permission.^[^ ^94^ ^]^ Copyright 2022, Wiley. (D) The EXAFS fitting curves of SA Fe‐N/S@CNF and SA FeN@CNF. Reproduced with permission.^[^ ^95^ ^]^ Copyright 2023, Wiley. (E) Schematic diagram of atomic structure evolution during the cycling process analyzed by XANES. Reproduced with permission.^[^ ^96^ ^]^ Copyright 2022, American Chemical Society. (F) The Cu K‐edge XANES spectra of Cu–Se DAs and the references (Cu SAs, CuSe, and Copper (II) phthalocyanine (CuPc)). Inset is the first‐derivative XANES curves of Cu K‐edge. Reproduced with permission.^[^ ^97^ ^]^ Copyright 2023, Wiley.

Besides, the advanced XAFS technique can also investigate the chemical reaction process/mechanism of the negative electrode materials in the cycling process of batteries, and then analyze the problems of low capacity, poor stability, and cycling performance caused by anode pulverization.^[^ ^93^ ^]^ To clarify the charge compensation mechanism of Na_0.6_Ni_0.25_Al_0.1_Mn_0.65_O_2_ (NAM01) under the reaction, the chemical states of transition‐metals from bulk to surface under different charge states were detected by ex‐situ XAFS with different detection depths. The Ni K‐edge and L‐edge XANES (Total electron yield, TEY, with a detection depth of ≈10 nm; Total fluorescence yield, TFY, with a detection thickness of ≈100 nm modes) spectra confirm this situation (Figure 3A–C). The XANES curves with TFY and TEY modes are reversible during charging and discharging, which indicates that the redox reaction of nickel is reversible. The Ni L‐edge spectra were divided into two peaks due to the crystal field splitting, and the oxidation state of Ni was reflected by the intensity ratio of the two peaks.^[^ ^94^ ^]^ In room temperature (RT) sodium–sulfur batteries, Bai et al.^[^ ^95^ ^]^ proposed that by adjusting the second‐shell coordination environment of single Fe atoms, atomically dispersed Fe‐N/S active sites are formed. EXAFS reveals the Fe‐N_4_S_2_ coordination structure with enhanced local electron density near the Fermi level (Figure 3D). This important work has opened up a new pathway for the complete conversion of polysulfides in RT Na‐S batteries. The evolution of electronic structure^[^ ^96^ ^]^ during the cycling process of TiO_2_ anode material in sodium‐ion batteries in the chemical reaction of salinization and desalination was clarified by XANES quantitively (Figure 3E). This work establishes a connection between the excellent electrochemical performance of carbon‐coated TiO_2_ nanoparticles in sodium batteries and the induced electronic and structural modifications during the sodiation and desodiation processes throughout cycling. Also, in a zinc–air battery, the local coordination environment of a dual‐atom catalyst containing adjacent Cu‐N_4_ and Se‐C_3_ active sites were investigated by in situ XAFS (Figure 3F). The right‐shift of adsorption position of Cu‐Se DAS indicates an increase in valence state. The extracted coordination parameters through EXAFS fitting, demonstrate that regulating the local coordination environment of Cu–Se is beneficial for improving the oxygen reduction reaction.^[^ ^97^ ^]^

### 3.2 SAXS for detecting nanoscale structure

SAXS can be used to study nano‐structure of materials, such as particle size, fractal, void, and orientation. The samples used for SAXS experiments can be solid and liquid and the thickness of the samples should be determined by considering their absorption of X‐rays. Two‐dimensional (2D) detectors, such as Pilatus, Eiger, or Mar165 CCD, were usually used to record the scattering patterns. The photodiode detector was coupled in the beamstop to record the intensity of the transmitted X‐ray beam. The length of the vacuum pipe, that is, sample‐to‐detector distance, can be adjusted to meet the needs of the samples to be tested according to the measurable maximum particle size (*d*_max_), which can be evaluated by the formula, *d*_max_ = *π*/*q*. Due to the transmission mode for the SAXS experiment, the material of the front and rear windows for in situ/operando battery cells needs a low absorption rate of X‐rays. After the cell was equipped properly between the vacuum pipe and 2D detector, the samples were exposed. The exposure time can be adjusted based on the maximum scattering intensity in order to obtain a good SNR data. SAXS also has some drawbacks, it cannot provide atomic‐level structural information, and the scattering patterns of particles and voids are the same and cannot be distinguished; the requirement of dynamic range of detector is high; the interference effects are not easy to deal with; and the data analysis method is not systematic. This section will discuss several typical applications of in situ SAXS, GISAXS, and SAXS/WAXS techniques in battery materials, aiming to illustrate the in situ SAXS techniques can effectively reveal the evolutions of nanostructures during the working processes of the batteries, which will lay an important foundation for the structural design and modulation of the battery.

**FIGURE 4:**
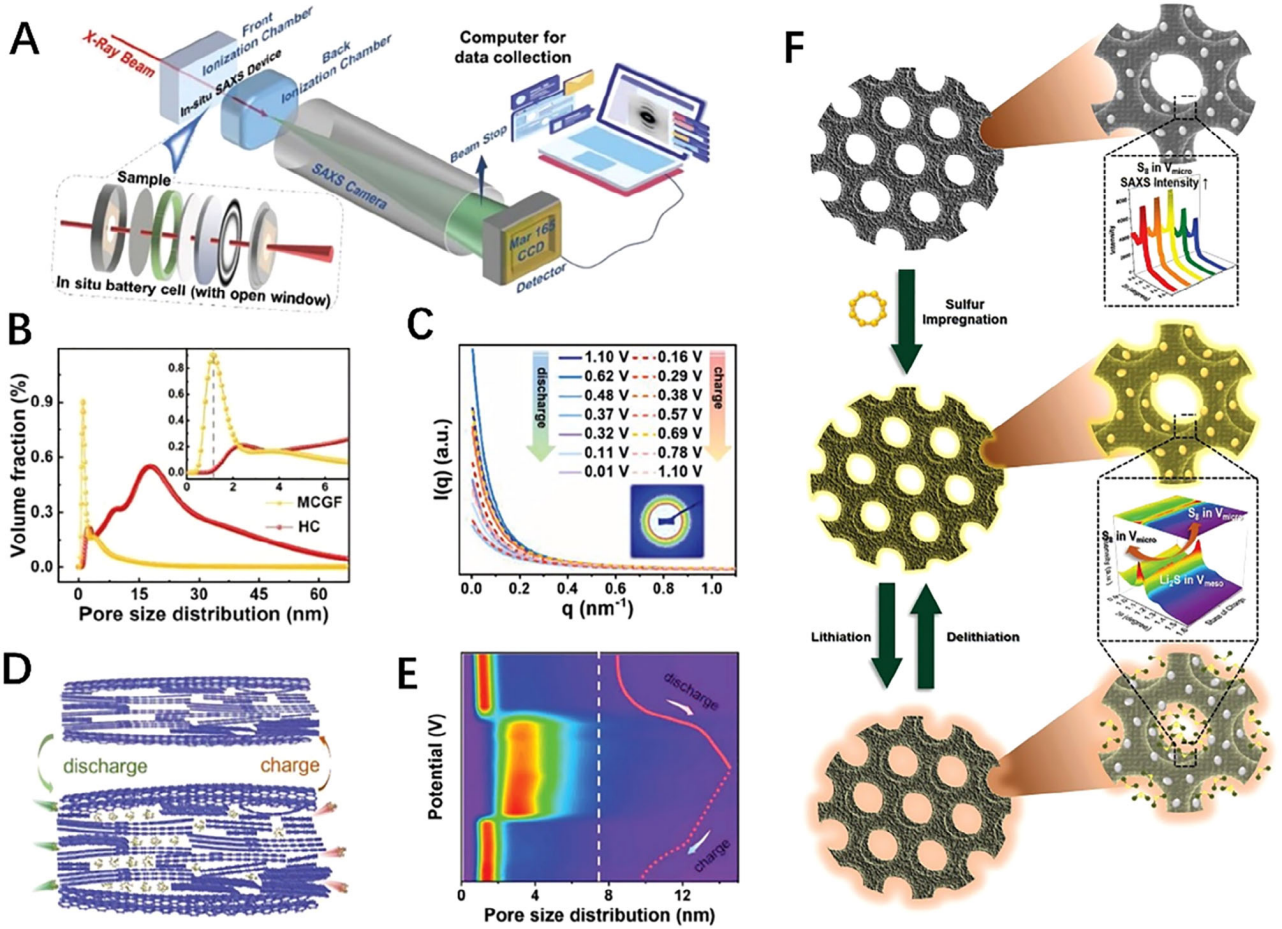
In situ small‐angle X‐ray scattering technique reveals the nanoscale size distribution. (A) Schematic diagram of the in situ SAXS during the electrochemical process. (B) Pore size distribution calculated by TBT method. (C) In situ curves of MCGF during the electrochemical process. (D) Schematic diagram of Na^+^ adsorption and desorption in the MCGF under electrochemical reaction. (E) Pore size distribution based on in‐situ SAXS data. Reproduced with permission.^[^ ^19^ ^]^ Copyright 2022, Wiley. (F) Schematic of S movement in OMC upon sulfur impregnation to lithiation and delithiation. Reproduced with permission.^[^ ^103^ ^]^ Copyright 2021, The Royal Society of Chemistry.

Though laboratory SAXS instruments (such as XENOCS, BRUKER, and ANTON PAAR) have been widely used in the study of battery materials,^[^ ^98^, ^99^, ^100^, ^101^ ^]^ SR SAXS has more advantages in investigating the structural evolutions^[^ ^102^ ^]^ due to the advanced capabilities of SR sources. Typically, the button cell with an aperture is placed on the path between the front and back ion chambers, and SAXS is imaged by Mar165 CCD (Figure 4A). The ion chambers can convert the photons into ionizing electrons. By measuring the number and energy of the ionizing electrons, the energy and intensity of the photons can be calculated. Liu et al.^[^ ^19^ ^]^ studied the pore structures and diffusion behavior of Na^+^ in microcrystalline graphite fibers (MCGF) in sodium‐ion batteries by in‐situ SAXS. The mesopore size distributions of hard carbon (HC) and MCGF were analyzed by applying the tangent‐by‐tangent (TBT) method to SAXS data (Figure 4B). The pore size of MCGF is in the range of 1–3.8 nm, which is smaller than that of HC. Therefore, the MCGF exhibits high crystallinity and graphitization, along with a significant presence of grain boundary voids and mesoporous cavities, which could help the excellent electrochemical performance and unique energy storage. The long‐periodic structural changes in MCGF were studied during a specific cycle of charge and discharge by in situ SAXS (Figure 4C). During the discharge process, the long‐periodic structure gradually increased, corresponding to the entry of Na^+^ into the grain boundary cavities. Correspondingly, the long‐periodic structure gradually decreased during the charging process, corresponding to the exit of Na^+^ from MCGF. This indicates that Na^+^ can be reversibly adsorbed/desorbed in the grain boundary cavities of MCGF (Figure 4D). The gradual increase/decrease in nanopore size during the discharging/charging process (Figure 4E) indicates the reversible adsorption and desorption of Na^+^ in the mesopores of MCGF, showing a new coadsorptive mechanism. The S migration in sulfur/ordered mesoporous carbon (S/OMC) during electrochemical reaction for Li–S battery has been investigated by in SAXS technique.^[^ ^103^ ^]^ There are two SAXS experiments conducted in this study. SAXS results reveal that the micropores primarily serve as sulfur reservoirs, while the mesopores are where the chemical reactions take place. The first experiment is sulfur impregnation which involves the deposition of sulfur into ordered mesoporous carbon with sulfur mass fractions ranging from 10% to 90%. As the sulfur loading reaches 20%, the scattering intensity reaches its maximum value due to the difference in electron density between the filled micropores and the unfilled mesopores. As the sulfur content continues to increase to 60%, the sulfur filling in the micropores reaches saturation, leading to a gradual decrease in scattering intensity due to the similarity in electron density between carbon and sulfur. The second SAXS test is about the movement of sulfur in 35% S/OMC material during the initial charge and discharge process. During the lithiation stage, the scattering intensity decreases sharply due to the movement of sulfur from micropores to mesopores and the formation of solid‐state lithium sulfide. In the subsequent delithiation stage, the scattering intensity recovers to near the initial intensity, as a large amount of reduced sulfur is transported back to the micropores (Figure 4F). In situ SAXS technique contributes to elucidate the mesopores are actual reaction site for sulfur species and the separability of the pores, which will add a key insight for the design of advanced carbon cathode material.

**FIGURE 5:**
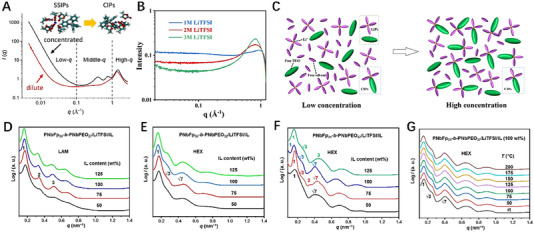
In situ small‐angle X‐ray scattering technique elucidate the nanostructure evolution. (A) SAXS data of the NaBPh_4_/PC electrolyte. Reproduced with permission.^[^ ^104^ ^]^ Copyright 2022, Elsevier. (B) SAXS data of the different LiTFSI concentrations. (C) Schematic diagram of LIPs, CIPs, free solvent, and TFSI^−^ in low and high‐concentration electrolytes. Reproduced with permission.^[^ ^106^ ^]^ Copyright 2023, American Chemical Society. One‐dimensional SAXS curves of the (D) PNbFp_30_‐*b*‐PNbPEO_27_/LiTFSI/IL, (E) PNbFp_30_‐*b*‐PNbPEO_32_/LiTFSI/IL, (F) PNbFp_31_‐*b*‐PNbPEO_37_/LiTFSI/IL complexes with various IL contents at ambient temperature, and (G) those of the PNbFp_31_‐*b*‐PNbPEO_37_/LiTFSI/IL complex with 100 wt % IL at different temperatures. Reproduced with permission.^[^ ^111^ ^]^ Copyright 2020, American Chemical Society.

The influence of electrolytes on different ionic batteries is reflected in the electrolyte's ion transport capacity (ionic conductivity), the ion de‐solvation energy at the electrode/electrolyte interface, and the ion transport capacity of the SEI produced by the decomposition of the electrolyte. These influences depend on the composition of the solvent and the properties of the solute, as well as the various additives in the electrolytes. SAXS was employed to characterize fluorine‐free electrolytes that solvation behavior of sodium tetraphenyl borate (NaBPh_4_) salt dissolved in different solvents of propylene carbonate (PC), 1,2‐dimethoxyethane (DME), acetonitrile (ACN), and tetrahydrofuran (THF).^[^ ^104^ ^]^ The interaction between salt and solvent can result in the formation of solvent‐separated ion pairs (SSIPs), solvent‐shared ion pairs, contact ion pairs (CIPs), or aggregates.^[^ ^105^ ^]^ The formation of these different types of ion pairs depends on the interaction between salt and solvent, as well as their relative concentration and temperature. The SAXS curve (Figure 5A) shows that in the low‐*q* region below 0.1 Å^−1^, the scattering intensity gradually increases due to the formation and increase of aggregates, and in the middle‐*q* region, the NaBPh_4_/PC electrolyte exhibits two characteristic peaks (peaks I and II), while only peak II is observed in other solvents (DME, CAN, and THF). In NaBPh_4_/PC electrolyte, the position of peak I composed of oriented stacked BPH_4_^−^ anions gradually shifts from 0.31 Å^−1^ to a higher *q* position of 0.4 Å^−1^ with the increase of concentration. Further molecular dynamics simulations based on SAXS data confirm the transition from SSIPs to CIPs with the increase in salt concentration. One of the main challenges in Li–S batteries is the shuttle effect. The concentration of electrolytes has a significant impact on the shuttle effect of lithium polysulfides (LiPSs). The electrolyte was prepared by dissolving lithium bis(trifluoromethane sulfonyl)imide (LiTFSI) in a mixture of 1,3‐dioxolane and 1,2‐dimethoxyethane(1:1,v/v).^[^ ^106^ ^]^ With the increase of LiTFSI concentration, the scattering peak in the high‐angle region gradually strengthens, indicating that a nanostructure is gradually formed in the electrolyte (Figure 5B). The formation of the nanostructure is that the free solvent molecules gradually decrease, and CIPs gradually form and increase from the low concentration of the electrolyte to the high concentration (Figure 5C). In addition, due to safety concerns with organic electrolytes in LIBs, polymer electrolytes based on block copolymers (BCP) have received a lot of attention.^[^ ^107^, ^108^, ^109^, ^110^ ^]^ The SAXS results show diffraction peaks with a scattering vector ratio of 1:2:3 for the PNbFp_30_‐*b*‐PNbPEO_27_/LiTFSI/IL complexes at ambient temperature (Figure 5D), indicating lamellar (LAM) nanostructures. The diffraction peaks with a scattering vector ratio of 1:$\sqrt{3}$:$\sqrt{7}$ confirm the transition of the nanostructures of the PNbFp_30_‐*b*‐PNbPEO_32_/LiTFSI/IL complexes from an LAM phase to a hexagonally packed cylindrical (HEX) phases when the content of the short poly(ethylene oxide) (PEO) in the BCP increases (Figure 5E). Also, the PNbFp_31_‐*b*‐PNbPEO_37_/LiTFSI/IL complexes form HEX nanostructures (Figure 5F). During the subsequent heating process, the rigid side chains within a block copolymer segment can achieve a stable liquid crystal phase, which enables the HEX nanostructure to be maintained up to 200°C (Figure 5G).^[^ ^111^ ^]^ In situ SAXS technique contributes to elucidate the self‐assembly structure evolutions of BCPs after doping of lithium salt and IL, which provide a beneficial guidance in potentially application of polymer electrolytes in LIBs at high temperatures.

### 3.3 XRD for resolving crystalline structure

XRD patterns reflect the long‐range ordered information of materials.^[^ ^112^ ^]^ XRD is an important technique to investigate the crystalline structure of electrode materials, such as lattice parameters, phase transition, strain, crystallinity, and grain size. Time‐resolved in situ XRD can real‐time survey the crystal structure changes of electrode materials during the electrochemical reactions. The samples used for XRD experiments can be solid powders, thin films and solutions. The in situ SR XRD experiments often conducted in high‐precision six‐circle diffractometer, Mythen or 2D detector (such as, Pilatus). The diffractometer adopts step‐by‐step scanning mode, while the Mythen and 2D detectors adopt exposure mode. The latter has a higher time resolution than the former, which can be up to milliseconds. Compared to XAFS and SAXS experiments, in‐situ/operando SR XRD measurement has a higher requirement for the in situ battery devices. Users can choose the appropriate experimental mode according to the change speed of the in‐situ battery reaction system. The in‐situ/operando XRD cell needs to be well matched on the diffractometer or curved Mythen detector, and well designed in order to fit the optical path. When conducting SR XRD experiment, researchers can increase reasonably the integrate time (for scanning mode) or exposure time (for exposure mode) to improve the SNR of data. Comparatively speaking, XRD technique also has some drawbacks, such as the inability to obtain meaningful diffraction data for electrode materials with amorphous or poor crystallinity and the small structures that exists only in trace amounts. It is also difficult to distinguish overlapping diffraction peaks. This section will discuss application examples of in situ XRD in battery electrode materials, aiming to illustrate that in situ XRD technique is of great significance for crystal structure research and performance modulation of electrode materials.

**FIGURE 6:**
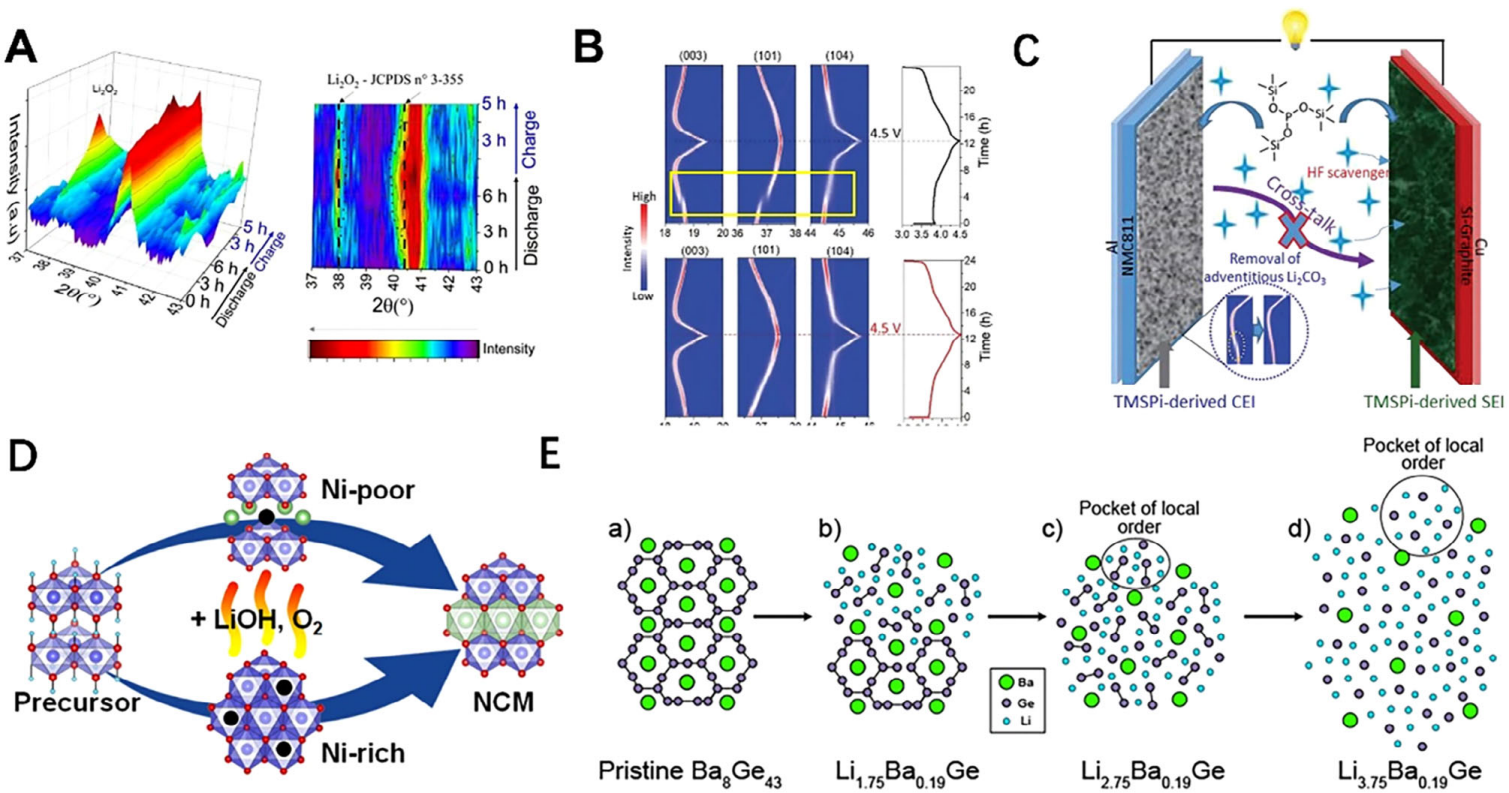
In situ X‐ray diffraction resolves the crystalline phase transitions in the process of electrochemical reaction. (A) Operando XRD characterization results of Li─O_2_ batteries assembled using LiBr as the redox mediator during the discharge/charge process. Reproduced with permission.^[^ ^59^ ^]^ Copyright 2021, American Chemical Society. (B) Operando XRD data and the corresponding charge/discharge curve of the NMC8111/Li battery during the first charge and discharge using the baseline electrolyte (top) and the TMSPi electrolyte (bottom), respectively. (C) Schematic diagram of NMC811/Si‐Gr full cell with TMSPi additive working mechanism. Reproduced with permission.^[^ ^115^ ^]^ Copyright 2020, American Chemical Society. (D) Structural transformation during the synthesis of NCM111. Reproduced with permission.^[^ ^116^ ^]^ Copyright 2023, American Chemical Society. (E) The schematic diagram of the electrochemical lithiation mechanism for Ba_8_Ge_43_. Reproduced with permission.^[^ ^117^ ^]^ Copyright 2020, American Chemical Society.

To track the reaction process of product formation and decomposition, Cremasco et al.^[^ ^59^ ^]^ conducted the complete operando discharge/charge cycle XRD characterization for the first time on an LiBr‐mediated Li─O_2_ battery with a high‐load electrode based on multiwalled carbon nanotubes (MWCNTs) (Figure 6A). During the discharge process, the diffraction peaks associated with Li_2_O_2_ crystalline phase gradually increased up to 7 h. In the presence of LiBr, the formation of Li_2_O_2_ nanocrystals began between 2 to 3 h after the start of discharge, or only after this time did the electrode accumulate a detectable amount of discharge product. Additionally, the contraction of the same peaks during the charging process also confirmed the decomposition of the product. The XRD results elucidated the reaction kinetics of Br^−^/Br_3_^−^ in the high carbon load electrode were elucidated, and confirmed that LiBr, as a redox mediator, indeed contributes to solving catalytic issues in the system, reducing charging plateau, and improving recyclability.

A new Li─Mn─O nano‐hybrid is used as a lithium‐ion battery cathode, and in situ SR XRD is used to discover it. The migration path of oxygen in the layered nano‐domain is blocked by the adjacent spinel nano‐domains with higher oxygen vacancy migration energy, effectively suppressing irreversible lattice oxygen loss under high potential, and improving the cycling stability of capacity and average voltage.^[^ ^113^ ^]^ The layered nickel‐rich oxide LiNi_0.8_Mn_0.1_Co_0.1_O_2_ (NMC811) has the advantage of high energy density and the disadvantage of rapid voltage and capacity decay. A deeper understanding of the structure and dynamic evolution of NMC811 will help to further understand its cycling behavior and mitigate its performance degradation. Therefore, SR XRD as a powerful tool was used to investigate the structural changes of NMC811 during electrochemical charge and discharge processes. In situ XRD tracked the evolution of the interlayer distance, which gradually increased before collapsing at a high state of charge (SOC). However, no two‐phase O_3_→O_1_ transition was examined at high SOC, indicating that this is not the main cause of degradation, but provides a new perspective for better understanding its rapid degradation mechanism.^[^ ^114^ ^]^ The cycling life of LIBs with NMC811 cathodes and high‐capacity Si‐Gr anodes is severely hindered by the continuous degradation of the electrolyte at the surface of the two electrodes. By introducing an electrolyte additive (tris (methylsilyl) phosphite (TMSPi)) to form a protective layer at the electrode/electrolyte interface to improve the electrochemical properties of the NMC811/Si‐Gr full cell. XRD data recorded during the first cycle of NMC811/Li batteries with baseline (top) and 2 wt% TMSPi electrolytes (bottom) (Figure 6B), demonstrate a significant difference (yellow dashed box) in the structural evolution and electrochemical curves of NMC811 electrodes prepared and cycled under the same conditions.^[^ ^115^ ^]^ This confirms that the addition of TMSPi can disrupt the formation of an indefinite Li_2_CO_3_ surface layer on the reactive surface of NMC811, allowing for the precipitation of solid solutions rather than a “two‐phase” reaction mechanism (Figure 6C). The synthesis of the lithium transition metal oxides, poor nickel (NCM111, LiNi_1/3_Co_1/3_Mn_1/3_O_2_) and rich nickel (NCM811, LiNi_0.8_Co_0.1_Mn_0.1_O_2_), was studied by in situ SR XRD.^[^ ^116^ ^]^ These two layered cathode materials have been developed of two completely different reaction mechanisms. The NCM811 will appear a rock salt type intermediate phase driven by high Ni content at ≈325°C, and the structure transforms a rhombohedral structure because of a sufficient amount of lithium ions and lowering steric constraints during the 2 h holding step at 500°C, while NCM111 only shows layered structure in the whole synthesis process (Figure 6D). By using XRD and pair distribution function (PDF) analysis, the electrochemical lithiation pathway of the type I clathrate Ba_8_Ge_43_ was studied, and the reaction mechanism was proposed (Figure 6E). In the initial Ba_8_Ge_43_, Ba is periodically distributed in the framework of Ge (Figure 6E‐a); after lithiation, the amorphous phase nucleates and grows (Figure 6E‐b); after every Ge insertion of Li, Ba_8_Ge_43_ is completely transformed into an amorphous phase composed of cavities, but there exists a local Li–Ge ordering between Ba atoms (Figure 6E‐c); upon full lithiation, an amorphous phase composed of Li–Ge is formed, with Ge atoms surrounded by Li (Figure 6E‐d). These results indicate that the Ba atoms in the clathrate structure dislocate the long‐range order of the Li–Ge clusters and kinetically hamper the nucleation and growth of the bulk crystalline phase.^[^ ^117^ ^]^

**FIGURE 7:**
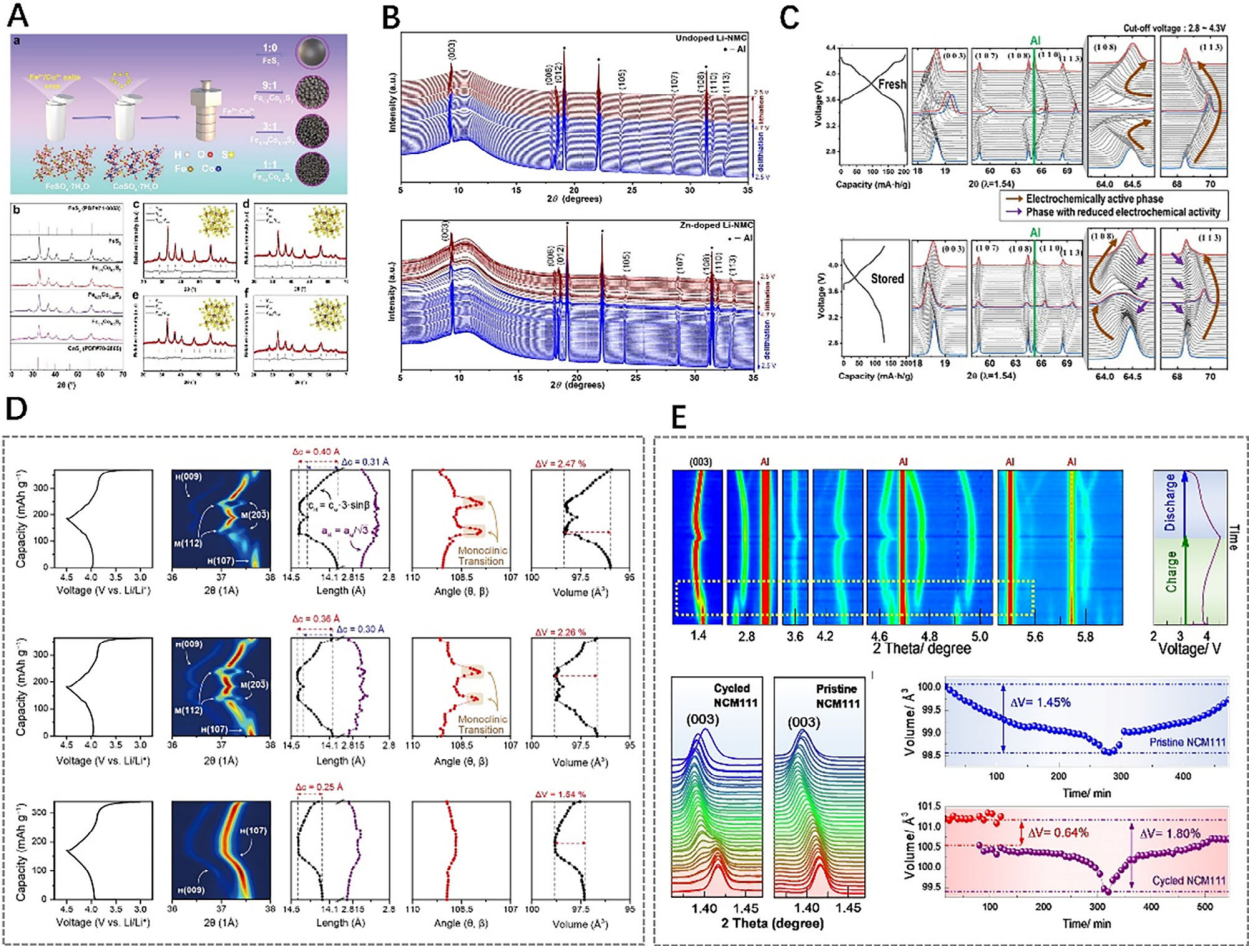
In situ/operando X‐ray diffraction study for revealing the crystal structures of some batteries when charging/discharging. (A) Schematic diagram of operando XRD tests for RMBs. Reproduced with permission.^[^ ^118^ ^]^ Copyright 2021, Wiley. (B) Operando synchrotron XRD patterns obtained during the delithiation and lithiation cycles @ C/10 with an upper cut‐off potential of 4.7 V (vs Li/Li+) for the undoped and Zn‐doped Li‐NMC‐based electrodes. Reproduced with permission.^[^ ^119^ ^]^ Copyright 2023, American Chemical Society. (C) Voltage profiles and XRD patterns were obtained by in‐situ XRD analysis using in‐situ half‐cells of the fresh NCA cathode (second cycle) and 60°C‐stored NCA cathode (cycled after the refreshing process) during charging and discharging at C/7. Reproduced with permission.^[^ ^120^ ^]^ Copyright 2022, Elsevier. (D) In situ synchrotron XRD analyses during the charge/discharge process. Reproduced with permission.^[^ ^121^ ^]^ Copyright 2023, American Chemical Society. (E) In situ XRD patterns of the cycled NCM111 during charging and discharging and the volumetric change of the cycled NCM111 lattice during a restarted cycle and a pristine NCM111 lattice. Reproduced with permission.^[^ ^122^ ^]^ Copyright 2023, American Chemical Society.

A rechargeable magnesium battery (RMB) electrolyte based on non‐nucleophilic phenolate magnesium complexes (PMC) is capable of reversible magnesium stripping/plating at a low overpotential of 84.3 mV at 1 mA cm^−2^. Co‐doping was introduced to prepare FeS_2_, Fe_0.9_Co_0.1_S_2_, Fe_0.75_Co_0.25_S_2_, and Fe_0.5_Co_0.5_S_2_, promoting the synergistic effect of Fe and Co. Figure 7A shows the preparation process and its XRD patterns of Fe_1−_ *_x_* Co*_x_* S_2_ samples, which verifies that the Co was doped in the samples. The operando XRD tests and the Rietveld refinement results confirmed that Co doping can extend the lattice and decrease particle size, which is conducive to cathodic reactions and promotes synergistic catalytic effects, and then improve electrochemical performance.^[^ ^118^ ^]^ To investigate the influence of Zn doping on the structural‐mechanical‐compositional integrity of Li‐TM‐oxide‐based cathode materials (Li‐NMC) after deep lithium removal, operando XRD was used to systematically study the changes of lattice parameters (Figure 7B) by Sharma et al.^[^ ^119^ ^]^ Typically, serious structural degradation occurs for Ni‐rich cathode materials when operated in high‐temperature condition, which often leads to severe performance degradation. Lee. et al.^[^ ^120^ ^]^ used SR‐XRD technique to investigate the influence of high temperature on the crystal and electronic structure of Ni‐rich cathode materials in the process of the electrochemical reaction (Figure 7C). In situ XRD technique observes that the bulk crystal structure of the Ni‐rich layered sample remains stable during high‐temperature storage, but changes occur in the surface structure and particle perfection. The significant growth of an insulating NiO‐like rock‐salt phase on the particle surface leads to asymmetric capacity loss in a way of charge decay. XRD helps elucidate the reasons for performance degradation of Ni‐rich cathode materials in high‐temperature condition, providing valuable insights for the development of heat‐resistant advanced Li‐ion batteries. Layered lithium cobalt oxide (LiCoO_2_, LCO) is a widely used cathode structure in LIBs, and its unstable phase transition during high‐voltage operation (≈4.5 V) remains a challenging issue. Several strategies have been put forward to emphasize this problem, but a clear understanding of their impact on LCO is yet to be established due to various underlying parameters such as particle size, shape, and dopant content. Bae et al.^[^ ^121^ ^]^ conducted in situ XRD analysis to further comprehend the effects of particle morphology and Mg doping on the structural changes of a series of LCO samples. It was confirmed that compared to plate‐like and spherical single‐crystal LCO samples, the influence of particle morphology and Mg doping effectively alleviated lattice strain. It also indicated a preference for Mg doping at the Co site (3b) instead of the Li site (3a) in the LCO framework (Figure 7D). This work gives a clear comprehension of Mg doping as a means to suppress the monoclinic phase transition. Besides, Zhang et al. studied the lifespan of LIBs during the charging‐discharging process.^[^ ^122^ ^]^ In situ SR‐XRD analysis uncovers that the harmful H_1_–H_2_ phase transition, which often occurs in the commercial NCM111 electrode during the recharge process after 90 cycles, is reactivated by the pausing process (Figure 7E). During the H_1_–H_2_ transition, the anisotropic lattice strain leads to mechanical fracture, which terminates at the inert NiO‐type rock‐salt phase on the particle surface. This suggests that discontinuous usage of rechargeable batteries is also a key factor in cycle life. This work provides a unique perspective on performance degradation in practical applications.

### 3.4 Combining techniques for multiscale structure detection

In fact, the structure of functional materials may be hierarchical during the electrochemical reactions and certain dynamic processes of electrode materials. The applied electrochemical factors can also induce materials transformations within hierarchical structures. The controllable synthesis and performance tuning of battery materials can be improved by tracking and capturing useful information on metastable phases and intermediates during the reaction process. Obviously, a single technique is difficult to meet all these requirements of hierarchical representation. The single‐structure characterization techniques will not satisfy the increasing needs of battery research. Undoubtedly, it is urgent and necessary to use in‐situ combination techniques to simultaneously obtain hierarchical information of dynamic processes. The combination of two or three techniques of in‐situ XAFS, SAXS, and XRD can detect the multiscale structural changes, which are capable of covering over the atom/molecular (local coordination structure), nanoscale (nanoparticle structure), and microscale (crystalline phase structure) dimension during the reaction process of the batteries. This section will discuss the important applications of combining techniques in the simultaneous acquisition of multiscale structures of the battery.

**FIGURE 8:**
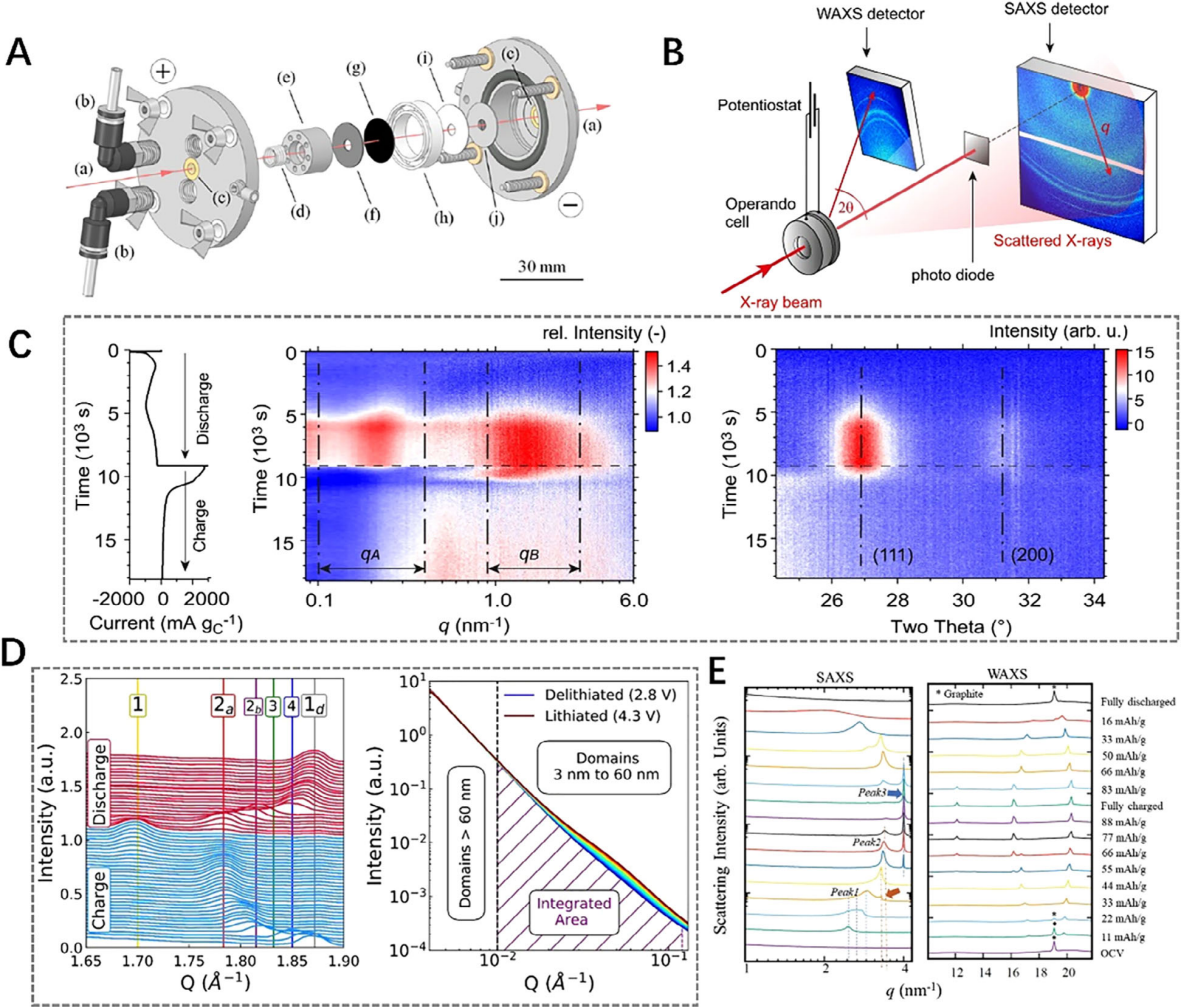
In situ/operando combined wide‐angle X‐ray scattering/small‐angle X‐ray scattering technique was used to investigate the nanoscale structural evolution of battery materials. (A) The test cell structure of Li–air battery for operando measurement. Reproduced with permission.^[^ ^125^ ^]^ Copyright 2022, American Chemical Society. (B) Sketch map of operando combined WAXS/SAXS measurements. (C) The relative SAXS and WAXS intensities change under specific currents versus time. Reproduced with permission.^[^ ^126^ ^]^ Copyright 2022, Springer. (D) The WAXS variations of the graphite lithiation stages (left), and the SAXS variations during charge from 2.8 to 4.3 V (right). Reproduced with permission.^[^ ^127^ ^]^ Copyright 2019, American Chemical Society. (E) Operando WAXS/SAXS technique was used to investigate the behavior of anion intercalation and reversibility in graphite. Reproduced with permission.^[^ ^128^ ^]^ Copyright 2023, Wiley.

The two SR X‐ray scattering of WAXS and SAXS, can provide atomic‐long‐period‐level and nano‐level structural information, respectively. The in situ combined WAXS/SAXS technique can provide simultaneously these‐levels information on the structural changes of electrode materials during electrochemical charging and discharging.^[^ ^123^ ^]^ However, the difficulty of this experiment lies in the need to design specific battery devices^[^ ^124^, ^125^ ^]^ that can be used for WAXS/SAXS combined testing when perform charging and discharging cycles. Designing and manufacturing a battery device that can be successfully applied to in situ SR tests is of great importance. Hatakeyama et al.^[^ ^125^ ^]^ designed a two‐electrode cell of an Li–air battery for operando WAXS/SAXS measurements (Figure 8A). The main material of the test cell was stainless steel. The cell is composed of (a) X‐ray path, (b) O_2_ tube joints, (c) X‐ray windows, (d) spring, (e) weight, (f) gas diffusion paper, (g) electrode, (h) guide ring, (i) separator, and (j) Li foil. Operando WAXS/SAXS experiments were conducted by Prehal et al.^[^ ^126^ ^]^ to investigate the nucleation, growth, and dissolution of solid deposits in an Li–S battery with a carbon black/S composite cathode and Li metal anode (Figure 8B). The SAXS and WAXS signals were recorded on separate detectors during the potentiostatic discharge/charge process, and the position of the detectors can be adjusted according to the range of data required. In some experiments, WAXS/SAXS data were obtained using the same detector, in which case the distance between the sample and the detector was relatively close.^[^ ^101^ ^]^ From the variations of SAXS and WAXS intensities as a function of time and scattering vector *q* during the discharge–charge process (Figure 8C), it can be seen that solid Li_2_S forms at about 5000 s. The (111) and (200) diffraction peaks in the WAXS plot also confirm the formation of Li_2_S crystalline grains with a mean size of about 6–7 nm. The SAXS peak in region *q*_A_ is attributed to Li_2_S crystalline aggregate with an average size of about 26 nm, and the *q*_B_ SAXS peak is attributed to a polysulfide structure comprised of Li_2_S*_x_* (2 ≤ *x* ≤ 4) with a mean size of ≈2.8 nm. However, these characteristic peaks in SAXS/WAXS disappear quickly due to the aggregates and Li_2_S*_x_* dissolving during the subsequent charging process. The LIBs with a‐Si/c‐FeSi_2_/graphite anode and LiNi_0.6_Mn_0.2_Co_0.2_ cathode was investigated by operando WAXS/SAXS combining technique during a 0.1C discharge/charge cycle^[^ ^127^ ^]^ (Figure 8D). In the WAXS profile, several Li*_x_* C_6_ phases can coexist simultaneously, and these 1_d_, 4, 3, 2_b_, 2_a_, and 1 phases are corresponding to C_6_, Li_≈0.167_C_6_, Li_≈0.22_C_6_, Li_≈0.33_C_6_, Li_0.5_C_6_, and LiC_6_, respectively; in the SAXS profile of charging process from 2.8 to 4.3 V, the scattering intensity exhibits the characteristic of Porod's law (*q* ^−4^) in the *q*‐range of low 10^−2^ Å^−1^, indicating that the size of the scattering particles in this region is larger than 60 nm. At the same time, the scattering intensity is a continuous change (blue to red) in the high *q*‐range from 10^−2^ to 10^−1^ Å^−1^, attributed to the volumetric swelling/shrinking of the silicon‐based phase during the lithiation/delithiation. Operando WAXS/SAXS technique was used to investigate the behavior of anion intercalation and reversibility in graphite.^[^ ^128^ ^]^ It can be observed that from SAXS curves (Figure 8E), peak 1 appears at about *q* = 2.2 nm^−1^ when the charging current reaches 11 mAh g^−1^ from the open circuit voltage (OCV), and then peak 1 moves towards higher *q* value, which is related to the intercalated AlCl_4_^−^ species. In the WAXS region, two weaker peaks appear. Subsequently, the graphite (002) diffraction peak at *q* ≈ 18.7 nm^−1^ disappears gradually with the charging process. When the charging current reaches 33 mAh g^−1^, peak 2 appears, and peak 1 disappears at the 44 mAh g^−1^ current. When the charging current is 55 mAh g^−1^, peak 3 appears at *q* = 4 nm^−1^ and two new diffraction peaks form at about *q* = 12 and 16 nm^−1^. The sharp and strong peak 3 is still present at *q* = 4 nm^−1^ when the battery is fully charged at 100 mAh g^−1^. The four diffraction peaks appearing in the WAXS range during the entire charging process indicate that the arrangement of the intercalated anions in the graphite structure is ordered. From the SAXS/WAXS plots, it also can be seen that the discharge process is completely symmetric to the charging process. The intercalation peak in the small‐angle range disappears after complete discharge, and the graphite diffraction peak reappears in the wide‐angle range, indicating the reversibility of the anion intercalation in graphite.

**FIGURE 9:**
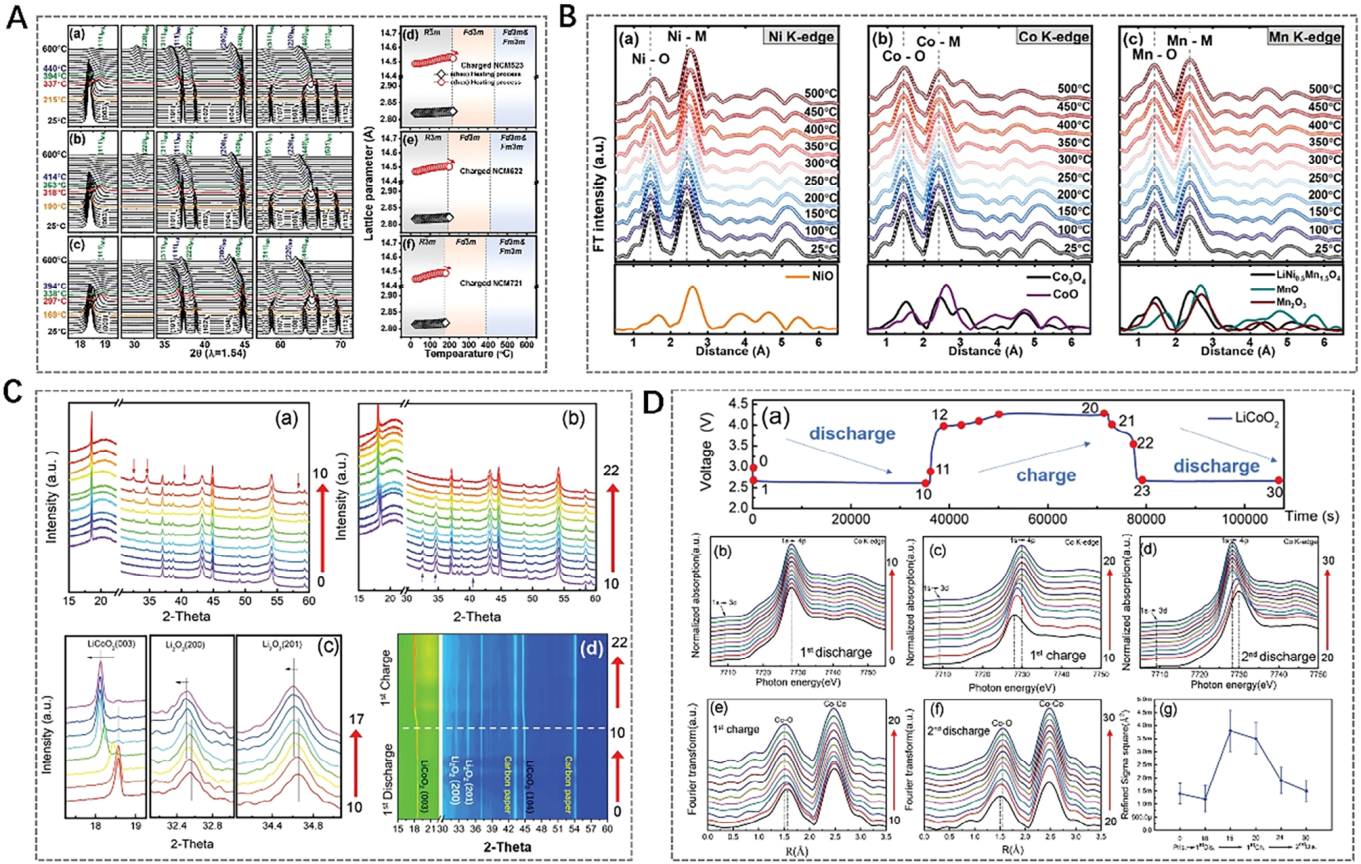
In situ/operando combined X‐ray diffraction/X‐ray absorption fine structure technique obtains the atomic neighbor structure and long‐range structure during the electrochemical reaction process. (A) In situ XRD patterns and change in lattice parameters and (B) Ni, Co, and Mn K‐edge Fourier transform magnitudes of k^3^‐weighted EXAFS spectra for the charged Ni‐rich cathode materials. Reproduced with permission.^[^ ^131^ ^]^ Copyright 2020, Wiley. In situ (C) XRD and (D) XAFS patterns of LiCoO_2_ based Li─O_2_ battery. Reproduced with permission.^[^ ^133^ ^]^ Copyright 2020, Wiley.

Besides, the XAFS/XRD combining technique was often used in batteries, capable of obtaining the atomic neighbor structure and long‐range structure, to investigate the electrochemical reaction process. Mullaliu et al.^[^ ^129^ ^]^ studied the reversible electrochemical lithiation process of potassium hexacyanocobaltate iron (FeCo) using XAFS/XRD combined technique, and confirmed that iron is the main electrical active site of FeCo. Due to its high energy density, thermal stability, and reversibility, Ni‐rich cathode materials are widely used in LIBs. There is an urgent need to understand the local and overall structural changes as a function of charging voltage, as well as their related effects on capacity decay. Quilty et al.^[^ ^130^ ^]^ studied the effects of voltage window on local coordination, bulk structure, and oxidation state of cells cycled at 3–4.3 or 3–4.7 V by simultaneous operando XRD/XAFS technique. Lee et al.^[^ ^131^ ^]^ used SR‐based XRD and XAFS to study cathode materials during heating, and proposed that thermal expansion and oxygen vacancies are new key factors affecting the thermal stability of charged Ni‐rich Li_0.33_Ni_0.5+_ *_x_* Co_0.2_Mn_0.3−_ *_x_* O_2_ (*x* = 0, 0.1, 0.2) cathode materials (Figure 9A,B). Operando XRD/XAFS technique confirmed that the high Ni content cathode material has a large thermal expansion, and oxygen vacancies are formed and accumulated around Ni ions until the spinel layer undergoes phase transition, which accelerate the migration of transition metals. The presence of both decreases the energy barrier of cation migration, thereby promoting the phase change of charged cathode materials under heating treatment. Aryal et al.^[^ ^132^ ^]^ explained the roles of Mn and Co in the formation of layered structure, charge balance, cationic mixing, and electrochemical performance using XRD/XAFS combining technique, from which the crystallographic information and electronic state can be obtained respectively, providing valuable information for the design of future Ni‐rich layered oxide cathodes. Gao et al.^[^ ^133^ ^]^ using LiCoO_2_ as an Li─O_2_ battery electrocatalyst to study the relationship between deep structure and performance with in situ XRD/XAFS technique (Figure 9C,D). The results indicate that the Co oxidation state makes changes and the electronic/crystal structure was regulated as well as the surface disorder degree, lattice strain, and local symmetry during the intercalation/extraction of Li^+^, all of which impact the catalysis activity. The in situ XRD/XAFS technique help to regulates the activity of Li─O_2_ battery catalysts by incorporating/removing alkali metal ions in traditional cathodes. Also, combined XAFS/XRD technique can reveal the storage mechanism of superior lithium storage (Guo et al.^[^ ^134^ ^]^) during the charging and discharging process. The operando XRD/XAFS technique contribute to develop various LIB anode materials with low conductivity and large volume changes. Other two combined techniques, such as grazing‐incident SAXS/XRD^[^ ^135^ ^]^ and SAXS/XAFS,^[^ ^136^ ^]^ were also be used as complementary structure evolution detection to reveal the reaction mechanisms in the field of electrocatalytic systems.

**FIGURE 10:**
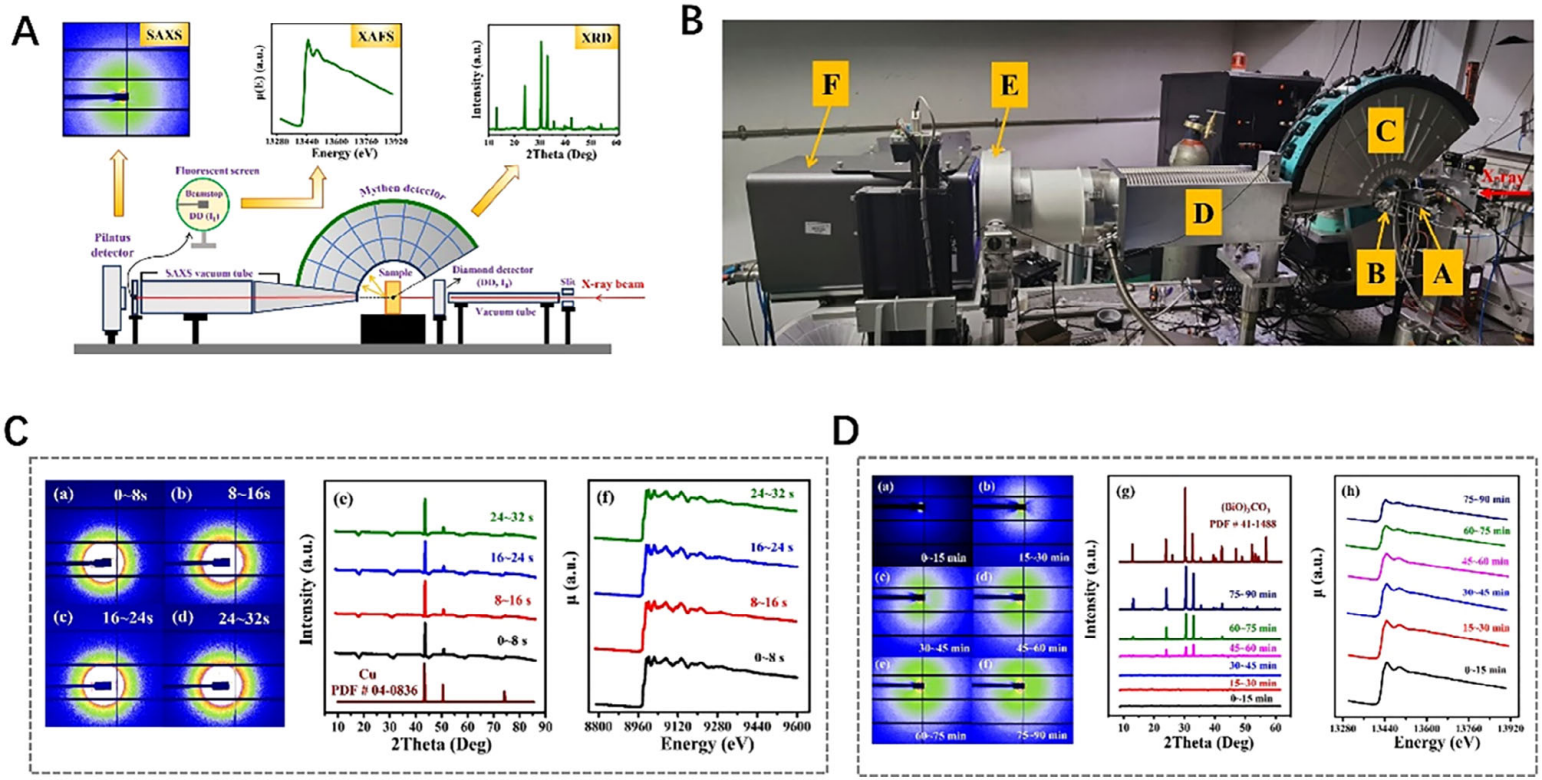
Novel in situ combined small‐angle X‐ray scattering/X‐ray diffraction/X‐ray absorption fine structure technique. (A) Schematic map of the SAXS/XRD/XAFS combined setup. (B) Photo of the SAXS/XRD/XAFS combined setup equipped in beamline 1W2B of BSRF. (C) SAXS (left panel) and XRD (middle panel) patterns as well as XAFS (right panel) spectra acquired by the SAXS/XRD/XAFS combined technique in two‐way energy‐scanning strategy with high‐frequency sampling scheme (100 kHz) for the Cu powder. (D) SAXS (left panel) and XRD (middle panel) patterns as well as XAFS (right panel) spectra acquired by in situ SAXS/XRD/XAFS combined technique in traditional one‐way energy‐scanning strategy for the hydrothermal synthesis of (BiO)_2_CO_3_ particles at temperature of 423 K and pressure of 3 MPa. Reproduced with permission.^[^ ^137^ ^]^ Copyright 2023, Springer.

It is well known that in scattering techniques such as SAXS, WAXS, and XRD, the incident X‐ray energy is constant, whereas in XAFS technique, it is energy‐dependent. When XAFS and SAXS/WAXS or XRD techniques come together, it is challenging to simultaneously acquire scattering and spectroscopic data because of the difference in incident X‐ray energies. The solution to overcome this difficulty is to alternately collect the scattering and spectra data, such as the above two combined techniques of XRD/XAFS and SAXS/XAFS. Actually, by reason of the alternating collection of scattering and spectral data, those combined techniques are only quasi‐simultaneous. Recently, a novel SAXS/XRD/XAFS combined technique (Figure 10A,B) was developed by Wu et al.^[^ ^137^ ^]^ at beamline 1W2B of Beijing Synchrotron Radiation Facility (BSRF) for simultaneous measurements for local atomic structure, nanoscale structure, and microscale structure at the same site in the reaction process for battery samples. Cu powder is tested with the SAXS/XRD/XAFS technique in Figure 10C. Four averaged SAXS, XRD, and XAFS data are acquired with two‐way energy‐scanning strategy in Figures 10C‐a–d and 10C‐e,f, respectively. A series of SAXS and XRD spectra are synchronously obtained in an XAFS scan. By energy calibration and statistical averaging, the series of SAXS and XRD spectra are unified into one SAXS or XRD pattern. Figure 10D shows (BiO)_2_CO_3_ particles form and grow in the hydrothermal reaction using the SAXS/XRD/XAFS technique. Six averaged SAXS, XRD, and XAFS data are acquired with one‐way energy‐scanning strategy in Figures 10D‐a–f, 10C‐g, and 10C‐h, respectively. The formation process of (BiO)_2_CO_3_ particles can be reflected through significant changes in SAXS and XRD graphs as well as XAFS spectra. It is believed that the newly developed SAXS/XRD/XAFS combined technique can be applied to study the time‐resolved processes, typically for the dynamic chemical reactions and particle growth process. Limited by the speed of mechanical motion, this SR combining technique on the first‐generation SR source can achieve a time resolution of the order of seconds. With the continuous innovation of technology and SR sources, the time resolution of the combining technique will reach a higher level. The ultra‐fast dynamic process of battery materials reaction will be monitored and the mechanisms inside the batteries will be well revealed. Anyhow, this current novel combined technique can disclose the structural evolutions from the atomic/molecular to nanoscale to long‐period micrometer scales by equipping with suitable in‐situ/operando sample environments.

## 4 SUMMARY AND OUTLOOK

In summary, this review highlights the important applications of SR XAFS, SAXS, and XRD as well as their combing techniques in revealing the internal electrochemical reaction processes and reaction mechanisms. It can be concluded that SR‐based techniques have become essential tools in resolving the various problems and challenges faced by batteries. This review only introduces the applications of the common and important three SR experimental techniques in battery materials research. However, the SR experimental techniques are not limited to these listed in this review, whose applications in batteries are also numerous and the application examples given in this paper are also only typical ones.

The advanced nature of elucidating multiscale structures makes the user group of SR techniques grow. From the point of view of techniques, as a large‐scale scientific facility, SR requires national investment to support its high construction and operating costs. The advancement of accelerator technology as well as the growth and demands of SR users have promoted the development of SR light sources. For example, the fourth‐generation High Energy Photon Source (HEPS) with world‐leading capabilities is being constructed and will be put into use in 2025, which can accommodate more than 90 high‐performance beamlines. After completion, it will become the SR light source with the lowest emission and highest brightness in the world. From the point of view of research or researchers, to resolve the key scientific problems in materials science, such as the battery/energy materials, and satisfy the characterization demands of high‐end users, the advanced SR experimental techniques have to be developed continuously. The SR characterizations of the material structure have developed from static to in‐situ dynamic evolution; the acquisitions of structure information have been obtained from single scale to multiscale. For example, except for the novel SAXS/XRD/XAFS combined technique mentioned above, the time‐resolved in‐situ WAXS/SAXS/USAXS combined technique is developing at beamline ID08 of HEPS, to acquire simultaneously the multiscale structure from atomic‐level to nano‐level to micro‐level. Besides, common characterization methods will be extended to various new experimental techniques such as coherence, transient, abnormal, and fine techniques, et al. Meanwhile, the combinations of SR technique and non‐SR technique are also the future development trend, such as SAXS/XRD/Raman, SAXS/XRD/IR, etc., which is conducive to an in‐depth understanding of chemical bonds, chemical composition, and molecular interactions of materials.

Battery systems are known as complex devices with numerous active components and competing physical and chemical interactions. We believe that the advanced SR techniques have a strong ability to gain a deep understanding of active materials. Here, it also should be noted that with the development of a high‐brightness SR source (for example, the luminous flux at a sample of beamline ID08 of HEPS is about 10^15^ phs s^−1^@12 keV), the damages of synchrotron X‐ray beam on samples should also be concerned for working batteries. Therefore, in order to gain true insight into the mechanism of the energy materials, it is also necessary for researchers to investigate the high‐flux beam damages or modifications on battery materials during in‐situ/operando SR experiments.

## CONFLICTS OF INTEREST STATEMENT

The authors declare no conflicts of interest.
